# Supplementary material for: Investigating the structural changes due to adenosine methylation of the Kaposi’s sarcoma-associated herpes virus ORF50 transcript
Source: PLoS Comput Biol. 2022 May 26;18(5):e1010150. doi: 10.1371/journal.pcbi.1010150 (PMC9176763; doi:10.1371/journal.pcbi.1010150)
Supplement: S1 Text — Section A: Additional detail for the methodology. A comment on why the exploration of energy landscapes is desirable, and how it is practically achieved. Details on how to judge sampling convergence in general are also included, and how these points apply to the present study. Section B: Additional structural information and more detailed structural representations. Section C: Description of the energy landscape for a shortened sequence, including the used methodology, a brief discussion of the results, and a disconnectivity graph of the free energy landscape. Section D: Additional analysis of the CV curves. Description of the third peak in the CV curves. Figure A: Initial structure obtained from RNAcomposer. The two-dimensional structure from experiment and the corresponding three-dimensional structure obtained from it. Figure B: Structural close-ups. Comparison of the apical loop, the upper helix H2 and the bulge for A, C, A⋆ and C⋆. Figure C: Free energy disconnectivity graph at 310 K for the shortened unmethylated loop. Figure D: Example structures for the high temperature transition. (PDF) [file pcbi.1010150.s001.pdf]

Supporting information:
Investigating the structural changes due to adenosine methylation of the Kaposi's sarcoma-associated herpes virus ORF50 transcript

Konstantin Röder[1,*], Amy M. Barker[2], Adrian Whitehouse[2], Samuela Pasquali[3,†]

1 Yusuf Hamied Department of Chemistry, University of Cambridge, Cambridge, UK
2 School of Molecular and Cellular Biology and Astbury Centre of Structural Biology, University of Leeds, Leeds, UK
3 Laboratoire CiTCoM, UMR 8038 CNRS, and Laboratoire BFA, UMR 8251 CNRS, Université de Paris, France

* kr366@cam.ac.uk, † samuela.pasquali@u-paris.fr

A Detailed methodology

A.1 Why we sample energy landscapes

A common problem in structural biology is encountered when we consider the structural polymorphism common, for example, in functional nucleic acids – the molecules in question exhibit a range of stable structures, but it is not clear how stable each of these configurations is, nor how fast a system can transition between states. This property has been described in detail in the literature, and occurs due to the broken ergodicity exhibited by biomolecules [1]. It is most prominently observed in the multiple time scales that biomolecular rearrangements span, and in the context of nucleic acids has also been described as kinetic partitioning.

As biomolecular structure and function are closely connected, it is desirable to understand these polymorphic structural ensembles in detail, and identify key structures and transitions between them. The theoretical underpinning that may be used to explain the molecular properties are based on the existence of the potential energy landscape. This energy landscape for each molecule contains the information needed to compute various properties of any molecular system [2]. As a result, detailed knowledge of the energy landscape will enable our understanding of the structural ensembles along the lines laid out above. This knowledge may be obtained in various ways, and in fact any method, whether experimental or computational, probes the energy landscape. Many methods do so implicitly, leading to constraints based on the topography of the energy landscape. For example molecular dynamics simulations are often used to study biomolecular systems, but as these computations retrace the dynamics of the molecular systems, the rare events studied remain slow, requiring enhanced sampling methods.

A complementary approach is provided by direct energy landscape explorations, i.e. our aim is to sample the energy landscape explicitly, and then in a second step derive observables such as thermodynamics and kinetics from the energy landscape.

The computational energy landscape framework [3–5] employed in this study follows this idea of direct energy landscape explorations. In this approach, the energy landscape is coarse-grained into local minima and transition states,[1] which connect the local

[1]Defined here as Hessian index-1 saddle points.

minima. The transition states are the highest-energy saddle points a system has to traverse between local energy minima. Sampling results in a database of local minima and transition states, allowing the computation of thermodynamic, kinetic and structural properties. The search for these stationary points can rely on geometry optimisation, which is independent of energy barriers, and hence independent of the characteristic time scales of the molecular system.

A.2 How we explore energy landscapes

Efficient exploration of the energy landscape relies on two factors. Firstly, an efficient way to locate transition states is required. These transition states not only should connect a set of minima, but furthermore we are seeking the low energy transition states, and they need to be physically acceptable. The low energy states will correspond to much faster transition rates, and hence locating low-energy transitions is required to converge the kinetic properties of the system under investigation. The physicality of the transition state is important in discrete modelling, such as discrete pathsampling (DPS), where we can go from one structure with the correct chirality and cis-trans stereoisomer to another such structure, through states where the chirality of a steric centre is inverted. Such transition states must be avoided to obtain reasonable transitions.

The second condition is that we need to find all low-energy minima. Importantly, while we can explore the energy landscape from a small subset of minima, the exploration will be faster and more efficient if our set of initial minima to be connected is as structurally diverse as possible.

A diverse set of minima might be located in a number of ways. For example, molecular dynamics simulations can be used to generate a diverse set of structures at high temperatures. Subsequent quenching of this set would then result in a suitable set of local minima to start exploration. A second option is to use available experimental structures, such as from X-ray or NMR experiments. Another option is to employ global optimisation, such as basin-hopping, to locate low-energy structures efficiently.

As no experimental structural data is available for this system, we used BH global optimisation [6–8] to obtain low energy structures. It should be noted that we were not aiming to find all low energy minima, but to locate a number of low energy minima to start the sampling. The sampling is fully parallel, and hence can make full use of modern computing architectures, and this approach therefore allows the use of multiple GPUs in parallel. As the basin-hopping is only used to seed the sampling, the convergence of the sampling is independent of the performance of the basin-hopping. The aim of the basin-hopping is therefore only to yield structures fairly low in energy that have some diversity. As such the exact choice of starting structures for basin-hopping and exact details of the searches will not change the sampled landscape, but will impact how efficient the landscape can be sampled.

Once we have located a suitable set of structures, we aim to connect structures by locating discrete paths between pairs of local minima [9, 10]. The discrete path is a series of minima and intervening transition states. In this case, we started our exploration using an *in*- and and *out*-configuration for an initial connection [11]. Once this path was connected, we used the UNTRAP [12], SHORTCUT BARRIER [12, 13] and CONNECTUNC [14] schemes in PATHSAMPLE[2] to converge sampling. UNTRAP removes artificial kinetic traps, SHORTCUT BARRIER finds lower energy barriers for already connected minima, and CONNECTUNC connects unconnected minima into the database.

Throughout, the doubly-nudged elastic band (DNEB) algorithm [15–17] was used with an initial linear interpolation. Transition state candidates were converged with

[2]See https://www-wales.ch.cam.ac.uk/PATHSAMPLE/

hybrid eigenvector-following [18], and minima located by following approximate steepest-descent paths on either side of the unique eigendirection of the transition state.

A.3 Sampling convergence

In an ideal case, a converged landscape would include all possible minima and transition states, however, such a level of sampling is unfeasible. This limitation mainly stems from the vast number of high energy minima, which we will likely not detect. Importantly, this limitation is likely to affect transitions between ordered and disordered structures, for example for melting transitions, but will not necessarily impact lower energy ordered to ordered transitions. As a consequence, we need to define what we mean by sampling convergence within the framework described. We may identify a number of possible answers to this question. Firstly, we can see convergence as the convergence of the landscape topography. In this case we refer to the fact that additional sampling is not affecting the number of funnels, nor their connectivity and no additional features appear.

The second possible definition is the convergence of thermodynamic properties. This criterion includes the correct location of the low energy minima, including the global minimum. This convergence is observed, when the addition of stationary points to the database is not altering the appearance of features in the heat capacity plot. Related to both of these points of view is the convergence of the relevant structural ensembles, i.e. that the structural ensembles have been found and their relative energies are described accordingly.

The final potential definition in this context is convergence of kinetic properties. The correct representation of dynamics is very difficult to judge, and without available comparison to experimental measurements nearly impossible. The methodology has been shown to faithfully reproduce the dynamics in other cases. A more detailed comment on the convergence is given in the next section.

A.4 A comment on the reported rates and equilibrium constants

An important question regarding this computational study is how accurate the reported equilibrium constant and rate constants are. We have two independent estimators to consider here.

Firstly, we use the NGT algorithm to calculate the kinetic properties of the system. NGT preserves the mean-first passage time of the system, which can be inverted to obtain the rate. The requirement on the kinetic transition network is therefore that we need to have the correct distribution of first passage times. An interpretation of this requirement is that we need to include all kinetically relevant paths to the product states in our calculation. This condition is likely met for low-entropy, folded states.

Given the large number of contacts preserved within the system, even for C and $C^{\star}$, we are likely in a regime where our description of paths is reasonable, as there are no unfolded or partially unfolded states involved in the transitions we observe.

Looking more closely at the heat capacity curves, which where obtained from the harmonic superposition approach from the minima located, we observe a clear separation between the second and the third peak for the m6A modified system, i.e. it is clear that the unfolding and structural changes are clearly distinguished processes, and the kinetic description is good. For the unmodified system, the peaks are much closer, and so there might be some error. This change would affect the rate constants somewhat.

The clustering within NGT is self-consistent. The process combines minima into states, while conserving the distribution of first passage times. The minima themselves

are converged for the states as the relevant thermodynamics (heat capacity features) are converged. As a result the error in our estimate would stem from the correct representation of the paths. More detail can be found in the cited literature.

Overall, in this study, we see convergence in the appearance of the energy landscape and in the thermodynamic properties as judged from the CV curves for low energy transitions. While we cannot definitely state that the dynamic properties have converged, it is important to consider the difference in the described energy barriers and rate constants. As the differences we describe are many orders of magnitude and the thermodynamics appear converged, any faster dynamics would not eradicate the stark differences between the methylated and unmethylated molecule.

B Structures details

In Figure A we report the sequence of the modelled system, the 2D structure already proposed in the literature, and the three-dimensional structure obtained from the 2D structure using RNAComposer. This structure is used to initiate path-sampling simulations for the native system and for the mutated system after having substituted A22 by m6A22.

In figure B we show the detailed 3D structures of the relevant regions undergoing changes in the four ensembles A, A*, C and C*, in relation to figures 5 and 6 in the manuscript.

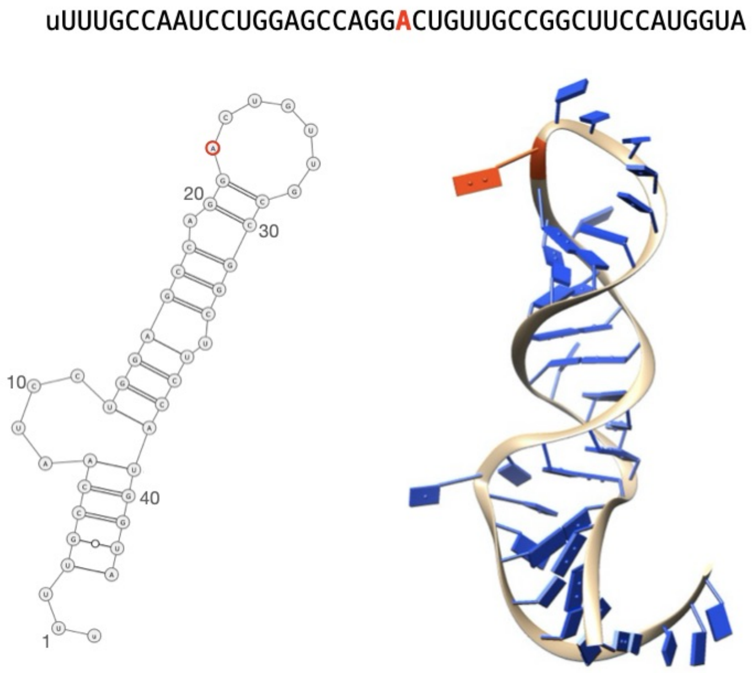

Fig A. Primary sequence, secondary structure from experiments and three-dimensional structure from RNAComposer. In red we highlight the modified base A22.

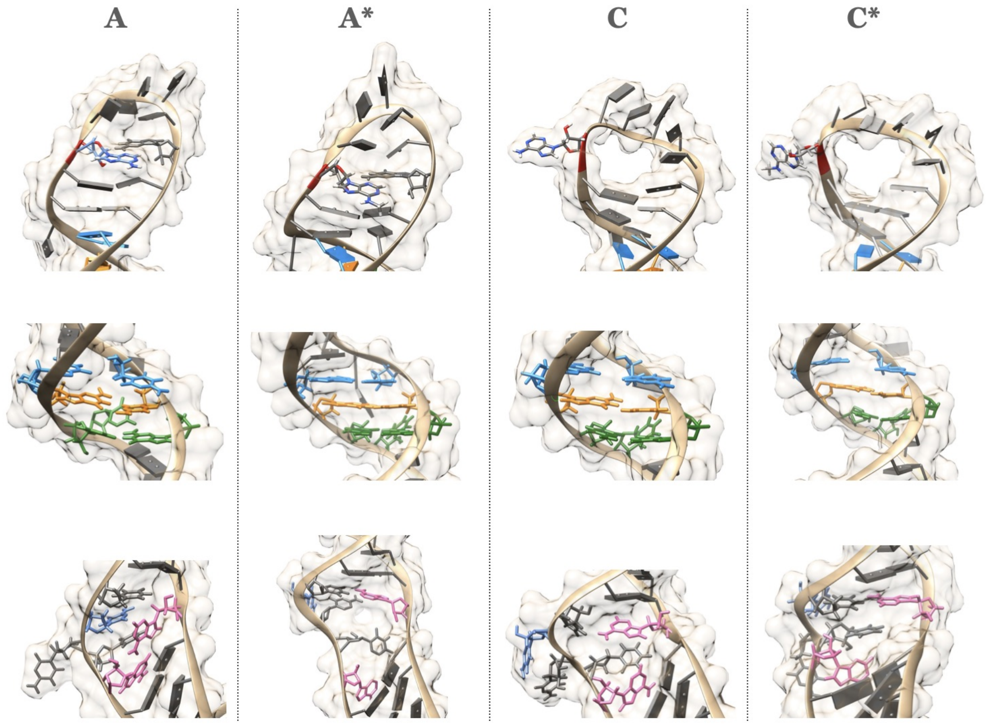

Fig B. Top: apical loop shown for the four ensembles. Nucleotide 22 is shown in atomistic detail and its backbone ribbon highlighted in red. Middle: upper helix (H2). Nucleotides G16, C33 and U34 are shown in dark green, the pair C17-G32 in orange, and nucleotides C18 and G31 in light blue. Bottom: central bulge (B). Nucleotides A8 and A38 are shown in pink, C11 is in light blue.

C Energy landscape for a shortened stem loop

While previous experimental work showed the requirement for the lower bulge in the regulation of SND1 recruitment [19], it is important to test that the energy landscape exploration for a shortened stem loop exhibits the same behaviour.

C.1 Methodology

The shortened sequence chosen contains the nucleotides 17 to 32, which includes A22, the apical loop and flanking nucleotides that form a stable stem on either side of the loop. We took the lowest energy minima for the *in*- and *out*-configurations from the unmethylated full stem loop energy landscape. We manually removed the unnecessary residues, and ran single point geometry optimisations on both minima. We then connected the minima in the same way as for the full sequence, and subsequently sampled the energy landscape in a similar fashion to the full sequence.

We used the same force field and parameter settings as for the full sequence.

C.2 Results and discussion

The free energy landscape at 310 K for the shortened sequence is shown in Fig. C, alongside example structures characteristic for the funnels on the energy landscape. Alongside a number of alternative loop structures for the *in*-configuration, we observe the *out*-configurations at much lower energies compared to the full length sequence. The unimolecular rate constants for the transitions are $4.043\times10^{4}\,\mathrm{s}^{-1}$ for the *out* to *in* transition and $2.327\times10^{4}\,\mathrm{s}^{-1}$ for the *in* to *out* transition, if we consider the structures that most resemble the structures we find in the full sequence. Considering all possible *in*-configurations, we find the transition rate constants as $4.046\times10^{4}\,\mathrm{s}^{-1}$ and $4.675\times10^{-1}\,\mathrm{s}^{-1}$, respectively.

In the case of the structures resembling the experimental structures most closely, these transition rate constants correspond to an equilibrium constant of 1.737.

It is clear from this data that the shorter sequence loses the energetic differentiation between the *in*-and *out*-configurations. This result is in line with experimental findings [19], and reinforces the interpretation that the lower bulge is required to differentiate between the *in*- and *out*-configurations, which is required for controlled functionality.

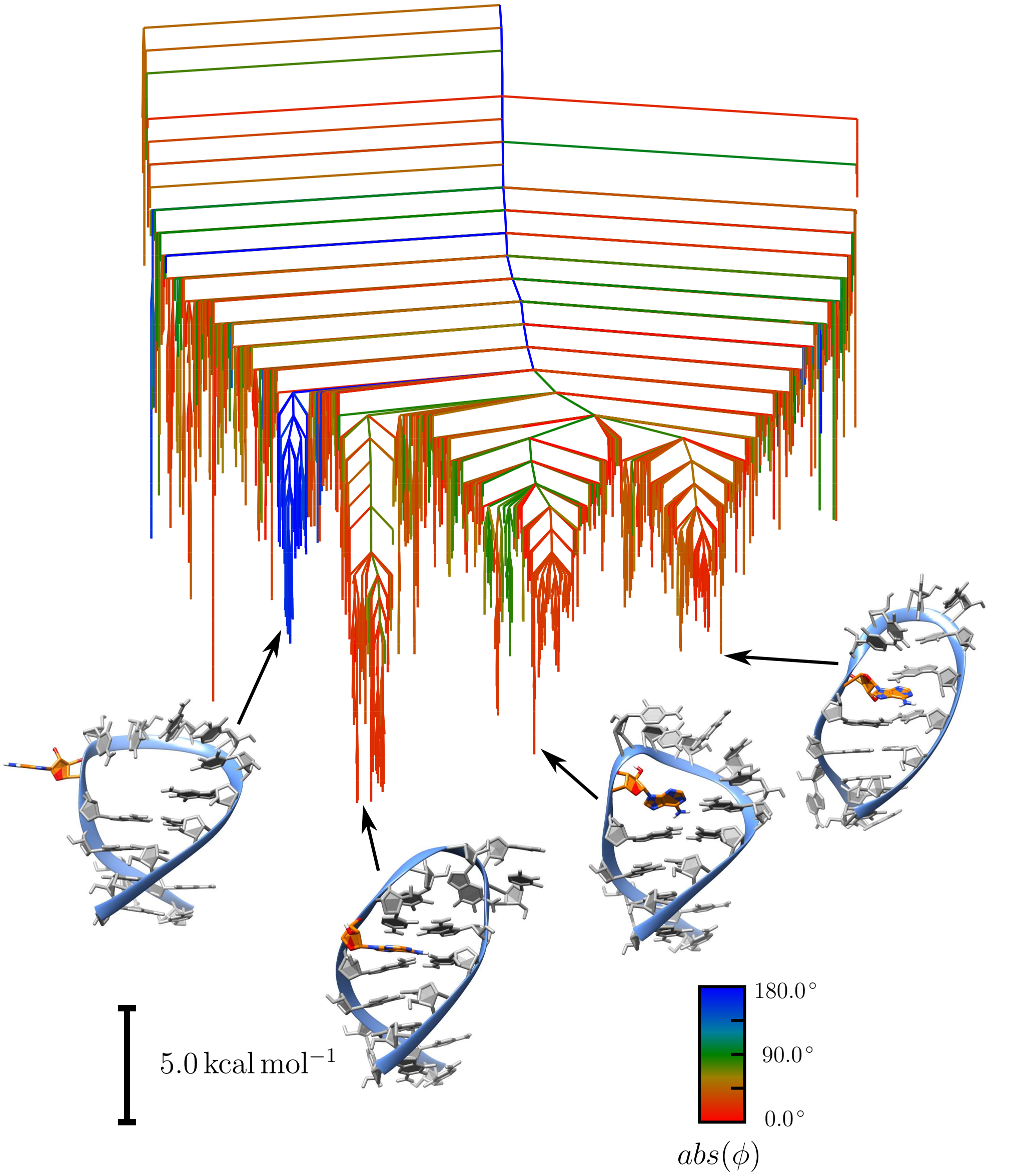

Fig C. Free energy disconnectivity graph at 310 K for the shortened unmethylated loop. The graph is coloured according to the absolute dihedral angle between A22 and the preceding nucleotide, where red are *in*-configurations and blue are *out*-configurations. The *out*-configuration is similar in energy to the *in*-configuration, and both the transition probability is increased compared to the stem loop and the transition rate is significantly higher.

D Additional analysis for CV curves

The analysis for the CV curves in the main manuscript focuses on the two peaks with the lowest transitions energies. This choice is based on the fact that $P1$ shows in both systems the transition between different lower stem arrangements, and $P2$ is the transition between *in*- and *out*-configurations.

In addition, there is another peak in both curves ($P3$). For the methylated system, this peak is well separated, while for the unmethylated system the peak forms a shoulder for $P2$. The transition for peak three corresponds to the loss of secondary and tertiary structure. This loss can be seen in the example structures shown in Fig. D. Especially the top part of the stem loop changes shape, visible in the loss of the tight helix observed at lower temperatures.

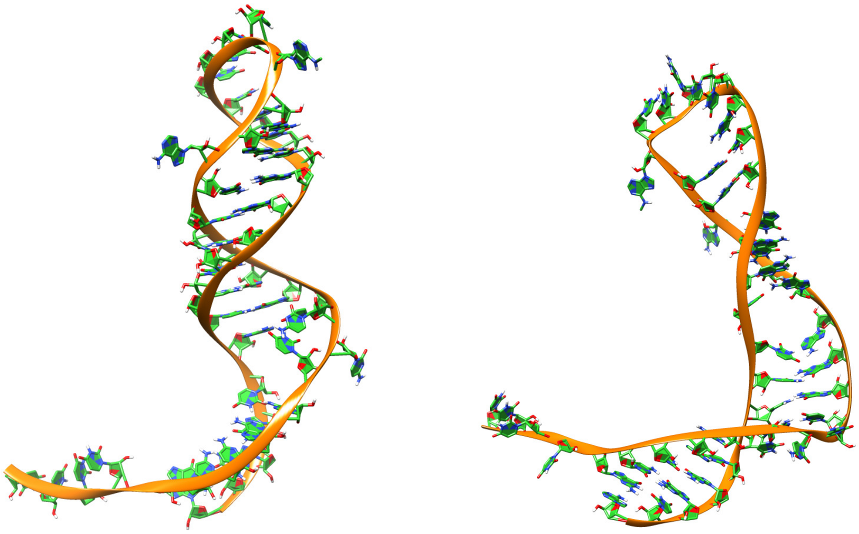

Fig D. Example structures for the high temperature transition ($P3$) for the methylated system. Left: Structures that are more likely occupied below the peak temperature. Right: Example structure which is more likely to be occupied above the peak temperature.

References

1. Wales DJ, Salamon P. Observation time scale, free-energy landscapes, and molecular symmetry. Proc Natl Acad Sci USA. 2014;111(2):617–622.

2. Wales DJ. Energy Landscapes. Cambridge: Cambridge University Press; 2003.

3. Joseph JA, Röder K, Chakraborty D, Mantell RG, Wales DJ. Exploring biomolecular energy landscapes. Chem Commun. 2017;53(52):6974–6988.

4. Röder K, Joseph JA, Husic BE, Wales DJ. Energy landscapes for proteins: From single funnels to multifunctional systems. Adv Theory Simul. 2019;2(4):1800175.

5. Röder K, Pasquali S. RNA modelling with the computational energy landscape framework. In: Ponchon L, editor. RNA Scaffolds. vol. 2323 of Methods in Molecular Biology. New York: Humana; 2021. p. 49–66.

6. Li Z, Scheraga HA. Monte Carlo-minimization approach to the multiple-minima problem in protein folding. Proc Natl Acad Sci USA. 1987;84(19):6611–6615.

7. Li Z, Scheraga HA. Structure and free-energy of complex thermodynamic systems. J Mol Struct. 1988;48:333–352.

8. Wales DJ, Doye JPK. Global optimization by basin-hopping and the lowest energy structures of Lennard-Jones clusters containing up to 110 atoms. J Chem Phys A. 1997;101(28):5111–5116.

9. Wales DJ. Discrete path sampling. Mol Phys. 2002;100(20):3285–3305.

10. Wales DJ. Some further applications of discrete path sampling to cluster isomerization. Mol Phys. 2004;102(9-10):891–908.

11. Carr JM, Trygubenko SA, Wales DJ. Finding pathways between distant local minima. J Chem Phys. 2005;122(23):234903.

12. Strodel B, Whittleston CS, Wales DJ. Thermodynamics and kinetics of aggregation for the GNNQQNY peptide. J Am Chem Soc. 2007;129(51):16005–16014.

13. Carr JM, Wales DJ. Global optimization and folding pathways of selected alpha-helical proteins. J Chem Phys. 2005;123(23):234901.

14. Röder K, Wales DJ. Energy landscapes for the aggregation of Aβ_{17-42}. J Am Chem Soc. 2018;140(11):4018–4027.

15. Henkelman G, Jónsson H. Improved tangent estimate in the nudged elastic band method for finding minimum energy paths and saddle points. J Chem Phys. 2000;113(22):9978–9985.

16. Henkelman G, Uberuaga BP, Jónsson H. A climbing image nudged elastic band method for finding saddle points and minimum energy paths. J Chem Phys. 2000;113(22):9901–9904.

17. Trygubenko SA, Wales DJ. A doubly nudged elastic band method for finding transition states. J Chem Phys. 2004;120(5):2082–2094.

18. Munro LJ, Wales DJ. Defect migration in crystalline silicon. Phys Rev B. 1999;59(6):3969–3980.

19. Baquero-Perez B, Antanaviciute A, Yonchev ID, Carr IM, Wilson SA, Whitehouse A. The Tudor SND1 protein is an m6A RNA reader essential for replication of Kaposi's sarcoma-associated herpesvirus. eLife. 2019;8:e47261.
