# Supplementary material for: Investigating the structural changes due to adenosine methylation of the Kaposi’s sarcoma-associated herpes virus ORF50 transcript
Source: PLoS Comput Biol. 2022 May 26;18(5):e1010150. doi: 10.1371/journal.pcbi.1010150 (PMC9176763; doi:10.1371/journal.pcbi.1010150)
Supplement: Attachment — Submitted filename: ReviewsPLOSComputBiol.pdf [file pcbi.1010150.s002.pdf]

Dear Prof Chen and Prof Elofsson,

We thank the reviewers for their feedback on our manuscript "Investigating the structural changes due to adenosine methylation of the Kaposi's sarcoma-associated herpes virus ORF50 transcript" (PCOMPBIOL-D-21-02101), and PLOS Computational Biology for reconsideration of the editorial decision after appeal.

In the following, we respond in detail to the reviewers' points. In addition to changes to the original manuscript, we have now added a document containing Supporting Material for publication.

Reviewer 1

Roder et al. investigate a stem-loop within ORF50 of KSHV by exploring the energy landscapes of the loop unmethylated and methylated at A22. They detail the structural differences between the unmethylated and methylated as well as between the in-configuration and out-configuration. The manuscript is well-written, and the science appears sound.

My main concerns are as follows:

1. As I was reading the manuscript, I was hoping to see the sequence and a general secondary structure representation of the stem-loop being discussed. Looking at Fig. 6 was helpful, but this seemed way too late in the manuscript. I realize that the secondary structure between the unmethylated and methylated can be different as well as the secondary structure between the in- and out-configuration. But, having some visual to set the stage early would be helpful.

   Authors' response: We have now included a figure in the supporting information (Figure S1) showing the sequence, the proposed 2D structure for the unmethylated system and the 3D structure obtained with RNAComposer, which constitute one of the seeding structures for simulations.

2. Three sets of BH runs were conducted. For one set, an unfolded structure was used as the starting structure. How did the structures resulting form the RNAComposer starting structure compare to the structures resulting from the unfolded structure? Would there be any benefit to starting with another unfolded (or random) structure?

   Authors' response: The basin-hopping was used to seed our sampling of the database. As a result, we used relatively short BH runs, and would generally speaking not expect convergence. Most of the structures resulting from the BH runs from the unfolded structure only exhibit a small number of formed base pairs. While these structures were included in the database, and will help to give an indication of the melting transition in the CV curves, they will have no effect on the reported transitions or equilibrium between the *in*- and *out*-configurations. In contrast, for the RNAcomposer derived structures we observe most if not all base pairs formed, and see the main variation in how they are arranged. We added a section in the supporting material that discusses the purpose of the BH runs in more detail.

   Given the purpose of the BH runs in this context we do not believe there is any benefit to seeding it with more structures. In other systems, however, especially if they are more polymorphic, running more BH runs and for longer is likely useful to increase sampling efficiency.

3. I was hoping to see more detailed structures showing the atom-atom interactions. For example, line 133 states that A22 "interacts with the surrounding nucleotides." Is it worth showing these interactions in a figure? Similarly, lines 235-239 discuss stacking in the unmodified system compared to the modified system and a different configuration of A22. Perhaps a detailed figure would help here too.

   Authors' response: We have now included a figure in SI (Figure S2) reporting the three-dimensional structural details of a representative structure of ensembles A, A*, C and C*. In the figure we zoom on the upper loop, the central region H1 and the bulge, where the main changes between the structures occur.

4. The authors conclude that the changes in the lower region and upper loop are connected (see "Changes in the bulge in the lower stem are required for out-configurations" section). What would happen if a smaller system was explored that consisted only of the upper loop (no bulge in the lower stem). If the conclusion is valid, no out-configurations would result. Can this be done to add validity to the conclusion?

Authors' response: In agreement with experiment, we would actually expect the opposite. The lower bulge is required to control the accessibility of the *out*-configuration, and removing the lower bulge should remove the bias towards the *in*-configuration. To confirm this hypothesis, which matches experimental results, we explored the energy landscape for a shortened sequence only containing the apical loop and some stabilising nucleoctides either end. The results are presented in the Supporting Information. In short, we observe more equal occupation probabilities and much faster transition rates, indicating that the lower bulge is indeed necessary to bias towards the *in*-configuration.

5. While reading the "The in-configuration in the unmodified and m6A-modified stem loop" section, it wasn't always obvious to me when the authors were referring to the unmodified versus the modified loop. Perhaps they could add some distinction there.

   Authors' response: We have edited this section to make it clearer whether we refer to the modified or unmodified system.

6. In Figs. 1 and 2, perhaps the authors could mention that A22 is colored red in the structures.

   Authors' response: We have added this fact to the figure captions.

7. I was a little confused by the drawings in Figs. 3 and 4. In Fig. 4, I understand the two structures in equilibrium as represented by P1. I do not understand the equilibria where one structure is drawn on one side of the arrow and two structures were drawn on the other side. Is the one structure in equilibrium with both structures?

   Authors' response: The transitions corresponding to the features in the heat capacity curves are between structural ensembles. The structures are chosen to be representative of the ensembles. The two structures were chosen to highlight the variation in the higher temperature ensembles. We have amended the figure captions to highlight this choice better, and also have added additional information in the Supplementary Material.

8. The authors didn't mention the shoulder in Fig. 3 and the additional peak in Fig. 4. Any ideas what they represent?

   Authors' response: These higher peaks are corresponding to the loss of key stacking interactions. As such they correspond to the high temperature behaviour, and representative structures are now provided in the SI.

9. In comparing Fig. 3 to Fig. 4, I did notice that the transition energy of P1 in Fig. 3 is very similar to the transition energy of P2 in Fig. 4. The same is true for the transition energy of P2 in Fig. 3 to the unlabeled peak in Fig. 4. Is there any significance to this? Similarly, in Fig. 4, why isn't the first peak unlabeled, the second peak P1, and the third peak P2 (making the transition energies of P1 and P2 very similar in both Figs. 3 and 4)?

   Authors' response: The matching in appearance is likely a coincidence. The features are labelled from low to high energy, with P1 the closest to 0. In fact, P1 and P2 in both graphs, respectively, correspond to similar transition. It is appropriate to compare P1 to P1 not because they are the lowest peaks, but because they are caused by the same change in the structural ensemble. The same is true for P2.

10. I was a little confused by Fig. 5. What do the pink and orange strips represent? I don't understand the y-axis for the first three panels (i.e. what are 1, 0.5, and 0 in relation to canonical base pairing?). I was also confused by the fact that, in the top panel for example, why there is a 1 on the top half of the plot and a 1 on the bottom half of the plot. Similarly, in the bottom plot, there are four C3'-endo labels on the y-axis. Why?

    Authors' response: We have included in the figure and in the caption a clearer explanation of the labels on the y-axis and of the meaning of the coloured stripes. Each panel in the figure is divided in a top and bottom part. On the top we report the valued computed for the unmethylated system while in the bottom part the values for the methylated system. This avoids too much overlap that would render the figure unreadable. The four panels give respectively: the average number of bases to which any given nucleotide is paired via a canonical interaction, computed on the ensemble of structures defining a funnel

(this value can vary from 0 to 1), the number of bases paired via non-canonical interactions (this value can vary from 0 to 2, considering all edges of the base), the number of stacking interactions of a nucleotide (this can vary from 0 to 2: one base above, one base below), and the pucker conformation. The pucker angle can vary from 0 to 360 degrees and it is then expressed according to the standard nomenclature reported in the literature and used in the RNA community.

11. In Fig. 6, can the blue and red frames be made darker and thicker? I also understood the "in-configuration" and "out-configuration" to refer to A22. In the caption for panel b, the authors use these terms to describe the lower bulge, which was confusing.

    Authors' response: We have updated the figures, such that the lines are clearer. In panel (b), we are referring to the lower bulge of these configurations, i.e. the left part of the panel shows the lower bulge in the *in*-configuration, and the right side the lower bulge in the *out*-configuration. We have updated the caption to make this more obvious.

Minor corrections:

1. Line 11 – "Stimulated" is misspelled.
2. Line 16 – "Transcriptome" is misspelled.
3. Line 26 – "recruitment the" should be "recruitment of the."
4. Line 32 – I am not familiar with DRACH. Is it an acronym?
5. Line 161 – "maybe" should be "may be."
6. Line 183 – "We can associated" should be "We can associate."
7. Line 205 – "Other key region" should be "other key regions."
8. Line 227 – "non-canonical interaction" should be "non-canonical interactions."
9. Fig. 5 – the "stacking" label is misspelled.

Authors' response: We have corrected these mistakes. For 4., a footnote has been added to clarify what a DRACH motif is.

Reviewer 2

This manuscript titled "Investigating the structural changes due to adenosine methylation of the Kaposi's sarcoma-associated herpesvirus ORF50 transcript" describes structural rearrangement in 43 mer stem-loop RNA due to m6A post-transcriptional chemical modification in its one of the bases. Authors have explored in and out configurations of A22 nucleotide in unmodified and 22m6A modified RNA structures. Authors have also revealed structural changes in stem-loops using the energy landscape framework. In order to obtain low energy structures author has used well known basin-hopping techniques and performed sufficient runs. Authors have also studied the kinetic transition network and its transition states using discrete path sampling and DNEB algorithm as well as hybrid eigenvector. Authors have studied rate constants and equilibrium constants of in and out the configuration of the unmodified and modified system, where they observed a significant number of out configurations in case of modified (m6A22) system.

Authors have well explained that how m6A modification of A22 base would result in exposed nucleotide outwards that would be key for the highly specific interaction with reader protein such as SND1. This manuscript is well written, and images were well organized and properly labelled.

However, I have the following major and minor concerns:

1. The WT and m6 modified RNA stem-loop structures studied here using computational methods are modelled structures. Are there any experimentally determined structure (3D or even 2D probing based structure) available to make sure that we are starting with the right structure? My worry is that depending on the starting structure (for example different arrangement of initial base pairs in the structure) we may

get a different result. On what basis is the starting structure of WT and m6A modified RNA different? Is there any experimental evidence for this available in the literature for the starting structures? Is there any experimental evidence as to which starting structure is more stable: WT vs m6A mutant?

Authors' response: As far as we are aware, only 2D structures have been reported by experiment, which we used to create 3D structures with RNAcomposer. The difference in these initial structures between WT and m6A is only in the modification of the nucleotide. Of course, as we then sample the structural ensembles the structures diverge somewhat, as described in the results section. Importantly, none of the changes we observe stem from differences in the initial structures.

Based on the modelling we would suggest that the WT *in*-configuration is more stable. This stability is mainly observed through the the absence of the binding in experiment.

2. Authors have used a previous study that reported that tudor SND1 protein is an m6A RNA reader essential for Kaposi sarcoma-associated herpesvirus as the justification for explaining the results here. That study however looked at m6A methylation and the role of SND1 as an m6A reader on a global scale using high throughput sequencing method. This study looked at things at a global scale on full-length RNA. The right study to compare the results would be when SND1 and the short stem-loop RNA (WT and m6A modified) would be used, showing the differences in binding. Is there any biochemical/biophysical evidence that showed that Tudor SND1 binds to the m6A modified stem-loop and not WT stem-loop RNA?

   Authors' response: There is experimental evidence, and we have edited the introduction to make this clearer. The cited work published in eLife (Baquero-Perez et al., 2019) contains such evidence. The work started with a global approach to identify m6A-methylated virual and cellular RNAs during KSHV infection. In Fig. 3 and the associated supplementary figures in the cited work it is clearly shown that SND1 binds the m6A modified stem loops, using native electromobility shift assays and recombinant SND1 protein.

3. In a result, authors related structural changes with heat capacity curves modified and unmodified systems and well-explained energy scale with Boltzmann population proportions for P2 peak of the modified system and observed a higher percentage of molecules compared to unmodified system. In figure 4, there is a third peak on the right-hand side of P2 peak, which is at a higher transition energy state. Can the authors explain this third peak?

   Authors' response: This third peak correspond to the loss of structural features, such as stacking and base pairing, and is associated with a transition form folded to partially folded structures. We have not fully sampled this transition, as we mainly focused on the ordered-ordered transitions that correspond to the *in* to *out* transition. We have added more detail on this in the Supporting Material.

Minor comments

1. Page 2 (introduction): Is it 'KSHV' or 'KHSV' in the sentence, "Several studies have demonstrated the KHSV transcriptome is heavily m6A methylated."?
2. Page 4 (last paragraph): the authors discussed the appearance of more distinct sub-funnels within in-configuration in the modified system due to changes in the lower stem-loop. However, there are alterations also present in the unmodified system but with no separate distinction of sub-funnels.
3. Table 1, Figure 1, 3, and 4: Is it "A23" or "A22"? It should be A22.
4. Page 5 (last paragraph line 183): "associated" can be "associate."
5. Page 6 (last paragraph line 224): The pairing is non-canonical (GU base pair) instead of canonical.

Authors' response: We have corrected these mistakes. With respect to 2., we believe the key feature is the appearance of distinct subfunnels in the modified system. We mention that there is some variation in the unmodified system.

Reviewer 3

Reviewer: The authors present a theoretical work addressing the effect of an m6A modification on the structural ensemble associated to a RNA stem loop. This modification is known to affect binding with a m6A reader (SND1). In this work, a significant effect of the methylation on the population of different states is observed. This effect is suggested to be responsible for the increased affinity of the methylated motif with SND1. The work is difficult to understand and, in my opinion, results are not correctly interpreted. As such, I think that publication is premature at this stage.

My main concern regards the results presented in Figures 1 and 2. The results are difficult to rationalize. Whereas I understand that the structure of the apical loop should be affected by the methylation, I don't understand how the structure of the lower part of the system could be correlated with the structure of the loop. This seems to be a key issue, since it is an important difference between the ensembles represented in Figure 1 and 2. The authors should provide an explanation. My suspect is that this is just a consequence of the random initialization of structures in the modified and non modified simulations.

Authors' response: The sampling of the landscapes is independent of the initial set of minima used, as long as we converge the sampling. We have added a detailed explanation about this aspect of the methodology to the Supporting Material. It should be noted that the initial set of minima will impact the efficiency of the sampling, as a better representation of all distinct configuration will significantly speed up sampling.

A second important point regarding this part of the review is that this relationship is not hypothesised or postulated by us, but was reported experimentally. We have also now added an exploration for a shortened, unmethylated stem loop, where, in the absence of the lower bulge, we do not observe the strong bias against the *out*-configuration.

Reviewer: The same problem emerges in Figure 6: how is the methyl group affecting the structure of the lower bulge (panel a)? There's no explanation for this, and I guess this is a random result.

Authors' response: This point is related to the above response. We report here the structural ensembles that we observe in the modelling. The results are in line with experimental findings.

Reviewer: A much more robust result could be obtained by using the same initial structures for the modified and not modified ensembles, just minimizing them separately with the modified / unmodified force fields. Are the authors doing this? As far as I understood, with the adopted procedure, it is extremely likely that randomness in the construction of the ensembles dominate the result.

Authors' response: As the structural ensembles between the two systems are different, it is not possible to simply use the same structures and minimise them. It should be noted that we have used the same initial structures and search procedure for both systems to initialise sampling. Importantly, to understand a system like this stem loop with multiple stable configurations, it is necessary to sample the entire structural ensemble. The energy landscape explorations provide one way of doing this, and are well established.

Furthermore, it is not clear what randomness the reviewer refers to here, and what this randomness in the structural ensembles means. The energy landscape is the fundamental object that describes the behaviour of any molecular system. Any method probes the energy landscape, though most approaches sample it implicitly, while we explicitly explore it. We have added these points to the Supplementary Material. Further information can be found in the reviews cited.

Reviewer: Line 74-80: "Three sets [...] landscapes." I am not sure I understand what the authors did. Are the unfolded structures (third set) modified or not? Isn't this choice leading to a different number of initial modified vs unmodified models? What's the rationale of this choice? Are the authors just building a large database and picking the 100 lowest energy structures? How many initial structures (generated with RNA composer) were used?

Authors' response: We have clarified this part of the methods section to make it clearer what was done. While it leads to a different number of initial minima in the database, the main effect of this is that the sampling times will differ somewhat. We have added a detailed discussion of this point in the supplementary material. The resulting databases are of similar size.

Reviewer: Line 118-120: Taken literally, the authors are claiming that the methyl group induces an energy shift of 12 kcal/mol. There is no explanation for such a large difference.

Authors' response: We are describing how the presence of the methyl group leads to structural rearrangements, which lead to a change in behaviour. In this context, a single hydrogen bond in water has an enthalpy of around 5 kcal/mol, and between water and amides are around 2 kcal/mol. The described changes in stacking, base paring and backbone configurations therefore can clearly sum up to such an energy shift. Again, the fact that the properties of the system are changed drastically by the methylation has been reported by experiment.

Reviewer: Line 133-138 and 293: I think there is a logical flaw here. The authors write the text as if the rates were a consequence of the free energy differences between the local minima. This is not correct: the rates are indeed a consequence of the differences between the local minima and the transition states. The rate between forward and backward transition rates then is the reason for the observed population. For instance, at line 293 the authors write that "the high stability of the in configuration [...] result in a very fast transition rate". This is logically incorrect.

Authors' response: While we discuss free energy and transition rates in this paragraph, we are not relating them. From the free energy differences, we point out that this will lead to different occupancy probabilities. We then discuss stability of the configurations. In this context we are talking about the depth of the free energy funnels, i.e. we consider a structure more stable if it sits in a deeper funnel. We agree that this was not clear from the original manuscript, and we have now added an explanation to clarify this part of the discussion.

Reviewer: Line 290: According to the prediction of the authors, the out configuration does NOT exist in practice in the unmodified stem loop (22-30 kcal/mol implies a negligible population). Table 1: I cannot understand how a difference of 22-30 kcal/mol (see line 118) or of 10 kcal/mol (see line 120) can result in the equilibrium constants reported in the table.

Authors' response: The equilibrium constants are calculated based on the forward and backward rates that are reported in Table 1 as well. Of course, it is possible to calculate the equilibrium constants based on thermodynamics, but the difficulty here is that there is a large number of states included in the equilibrium. For the kinetics, we use the NGT algorithm, which accounts for the number of possible states within the *in* and *out* configurations, as well as for intermediate states. The approach the algorithm follows is to reduce the number of states iteratively while reweighting transition probabilities, which preserves the complex multistate kinetics.

If we assume a simpler two configuration system with energy differences of 10 and 22 kcal/mol, respectively, the equilibrium constants are $1.1 \cdot 10^7$ and $3.2 \cdot 10^{15}$, as calculated with $\Delta G = -RT \ln(K)$. Clearly, the correct treatment of the multistate nature of this transition network is important.

Reviewer: Line 158 "A useful way". I cannot see how this representation can be useful. As far as I understand, this heat capacity is not related to anything that can be measured experimentally.

Authors' response: The graphs shown are related to the heat capacity. The main problem in relating our reported figures to experiment is that we ignore anharmonicity and the solvation is not represented properly. In addition, the high energy structural ensembles are not sampled fully, as they are not relevant for the studied low-energy transition.

Within these limitations the represented heat capacities are qualitatively correct. The importance within this study is that the clear difference in the two systems relates to the accessibility of the different states. In short, for the modified system we expect both configurations top be accessible, while for the unmethylated system we only expect the *in*-configuration to be relevant at biologically relevant temperatures. These qualitative differences in the heat capacities might be observed in experiment, whereas the very slow kinetics is less likely observable.

Reviewer: Figure 5: there's no explanation for the labels in the last panel. Why is C3'-endo repeated?

Authors' response: We have included in the figure and in the caption a clearer explanation of the labels on the y-axis and of the meaning of the coloured stripes. See also the answer to ref 1. Puckering values run from 0 to 360 degrees. Angles in range from 0 to 20 degrees are classified as C3'-endo. The label is repeated to highlight the periodicity of the values adopted (it's the same as identifying 360 with 0).

Reviewer: Lines 364-374: As far as I understand from this text, the experiment suggests that there is a conformational change driven by methylation. It does not suggest that the lower bulge should be affected by the methylation. So, this finding, which is puzzling (see above), is not validated in any way.

Authors' response: The lower bulge is required as shown by experiment. We therefore aim to identify the links between the changes in the apical loop and the lower bulge. We have now added the results for exploring

the energy landscape for a short loop. In this system, where there is no lower bulge, we observe a loss of the bias towards one configuration, adding further validation.

Finally, the fact that we observe no *out*-configuration that has the same lower bulge configuration as the gloabl free energy minimum in both cases shows that such a change is very likely required.

Minor issues:

Line 11 Typo (”Stimualted”)
Line 98: I would add a reference rather than showing the AMBER keyword (igb=2)
Line 201: ”were” -> ”where”
In several places, the authors write A23 instead of A22 (can be found with text search).
Figure 5: staking -> stacking
Figure 5, caption: ”Barbnaba” -> ”Barnaba”

Authors’ response: We have corrected these mistakes and added the missing reference.

Additional questions

Have the authors made all data and (if applicable) computational code underlying the findings in their manuscript fully available?

Reviewer 1: Yes
Reviewer 2: Yes
Reviewer 3: Yes

PLOS authors have the option to publish the peer review history of their article. If you choose “no”, your identity will remain anonymous but your review may still be made public.

Do you want your identity to be public for this peer review?
Reviewer 1: No
Reviewer 2: No
Reviewer 3: No
