# Supplementary material for: Investigating the structural changes due to adenosine methylation of the Kaposi’s sarcoma-associated herpes virus ORF50 transcript
Source: PLoS Comput Biol. 2022 May 26;18(5):e1010150. doi: 10.1371/journal.pcbi.1010150 (PMC9176763; doi:10.1371/journal.pcbi.1010150)
Supplement: Attachment — Submitted filename: ReviewsPLOSComputBiol_round2.pdf [file pcbi.1010150.s003.pdf]

Dear Prof Chen and Prof Elofsson,

We thank the reviewers for their feedback on our manuscript “Investigating the structural changes due to adenosine methylation of the Kaposi’s sarcoma-associated herpes virus ORF50 transcript” (PCOMPBIOL-D-21-02101).

We have addressed all the editing changes suggested by reviewer 1. Please find our responses to the queries from reviewer 3 below.

Reviewer 3: The authors replied to my questions. It turned out that most of my doubts were related to unclear explanations. To be fair, I still do not understand some of the presented results and I would encourage the authors to clarify them for the benefit of the readers:

1. When comparing Fig 1 and Fig 2, I see two major effects: m6A stabilizes C wrt B and m6A stabilizes A wrt B. The former makes sense (I still do not understand how the change can be this large, but I trust the authors). The second I suspect is due to the randomness of the basin hopping algorithm. If this is correct, please comment this in the caption.

2. If I understand correctly, the discrepancy between the large free energy differences and the relatively small Keq (Table 1) is a missing entropic effect associated to configuration count. If this is correct, please explain this in more detail in the paper. Readers will implicitly associate free energies with populations and macrostates, whereas here the authors are reporting free energies for micro states, and the number of these states is different in different macro states. Please also report an estimate in the count of the number of states that could justify this discrepancy.

3. Related to the previous point, is the horizontal density of points in Figs 1 and 2 uniform? If so, can I deduce that basin hopping is NOT able to report the correct number of states that would be required to correctly compute populations of macro states? Again, a comment on this issue would help the reader.

4. Finally, one small issue that I didn’t mention in my first review is that it might be interesting to know if m6A is in syn or anti conformation in the in- and out- state. In general, m6A is expected to be anti when WC paired and syn when non WC paired (see e.g. https://pubmed.ncbi.nlm.nih.gov/25611135/). Here, in the in-state it is forming a non canonical pair with G28 (Fig. 6c). It would be interesting to know if this pairing requires A22 to flip to the least stable anti conformation or not. The analysis should be straightforward and the result could be useful.

Authors’ response:

1. We believe that these changes stem from the alterations in the stem loop as a result of the methylation, in particular in helix 2 and the bulge configuration. The changes are described in the section “m6A22 destabilises the central helix H2 and alters the bulge structure”. We have now added a sentence to make it clear that we believe these changes are responsible for the difference between $A^{\star}$ and $B^{\star}$. In Fig. 2 it can be seen that a key change between $A^{\star}$ and $B^{\star}$ is the relative orientation of H1 and H2, which results from changes in the bulge. These changes are related to the changed base pairing as shown in Fig. 6. For the reference to randomness, please see the next two points.

2. Within this study we employed the NGT algorithm. NGT preserves the mean-first passage time, which we invert to get a rate. To obtain the correct rates, we therefore require that the kinetic transition network has the correct distribution of first passage times. The current understanding of this requirement is that we need to include all kinetically relevant paths to the product states in our calculation. This condition is likely met going to low entropy states.

   Given the large number of contacts preserved within the system, even for C and $C^{\star}$, we are likely in a regime where our description of paths is reasonable, as there are no unfolded or partially unfolded states involved in the transitions we observe.

   There is a caveat to this argument. Looking more closely at the heat capacity curves, which where obtained from the harmonic superposition approach from the minima located, we observe a clear separation between the second and the third peak for the m6A modified system, i.e. it is clear that the unfolding and

structural changes are clearly distinguished processes, and the kinetic description is good. For the unmodified system, the peaks are much closer, and so there might be some error. This change would affect the rate constants somewhat.

The clustering in itself is self-consistent. The process combines minima into states, while conserving the distribution of first passage times. The minima themselves are converged for the states as the relevant thermodynamics (heat capacity features) are converged. As a result the error in our estimate would stem from the correct representation of the paths.

The details of all these methods can be found in the cited literature, but we have nonetheless added more detail to the supporting material to make these points clearer.

3. As discussed in the previous responses to reviewers, in the methodology section and the supporting material, basin-hopping is not a sampling but a searching method, and it was employed as such. The sampling of the energy landscape employed discrete pathsampling (DPS). To judge convergence we need to rely on various observables, as described in the supporting material. For the described states we can be reasonably confident of convergence as the heat capacity features appear converged. DPS performs well for such states, but due to the nature of unfolded states it will be difficult, if not impossible to converge high entropy states. Within the computational energy landscape framework, another method, basin sampling, was developed to deal with such situations, but as unfolding is unlikely to play an important role in this case, we did not employ it here.

   Regarding the distribution of states, we would not expect it to be uniform, for example see Röder et al., *Adv. Theory Simul.*, 2019 (ref. 14).

4. We see m6A22 in its *syn*-configuration, and the base pairing is formed through the sugar edge. We have added this detail to the results section.
